# Coronary artery assessment in Kawasaki disease with dual-source CT angiography to uncover vascular pathology

**DOI:** 10.1007/s00330-019-06367-6

**Published:** 2019-08-19

**Authors:** D. van Stijn, R. N. Planken, M. Groenink, G. J. Streekstra, T. W. Kuijpers, I. M. Kuipers

**Affiliations:** 1 Department of Pediatric Hematology, Immunology and Infectious Diseases, Emma Children’s Hospital, Amsterdam UMC, University of Amsterdam, Amsterdam, The Netherlands; 2 Department of Radiology and Nuclear Medicine, Amsterdam UMC, University of Amsterdam, Amsterdam, The Netherlands; 3 Department of Cardiology, Amsterdam UMC, University of Amsterdam, Amsterdam, The Netherlands; 4 Department of Pediatric Cardiology, Emma Children’s Hospital, Amsterdam UMC, University of Amsterdam, Amsterdam, The Netherlands

**Keywords:** Kawasaki disease, Pediatrics, Coronary artery disease, Computed tomography angiography, Echocardiography

## Abstract

**Background:**

Kawasaki disease (KD) is a vasculitis with formation of coronary artery aneurysms (CAAs) that can lead to myocardial ischemia. Echocardiography is the primary imaging modality for the coronary arteries despite limited visualization. Coronary angiography (CAG) is the gold standard yet invasive with high-radiation exposure. To date however, state-of-the-art CT scanners enable high-quality low-dose coronary computed tomographic angiography (cCTA) imaging. The aim of our study in KD is to report (i) the diagnostic yield of cCTA compared to echocardiography, and (ii) the radiation dose.

**Methods and results:**

We collected data of KD patients who underwent cCTA. cCTA findings were compared with echocardiography results. In 70 KD patients (median age 15.1 years [0.5–59.5 years]; 78% male; 38% giant CAA), the cCTA identified 61 CAAs, of which 34 (56%, with a *Z* score > 3, in 22 patients) were not detected by echocardiography. In addition, the left circumflex (aneurysmatic in 6 patients) was always visible upon cCTA and not detected upon echocardiography. Calcifications, plaques, and/or thrombi were visualized by cCTA in 25 coronary arteries (15 patients). Calcifications were seen as early as 2.7 years after onset of disease. In 5 patients, the cCTA findings resulted in an immediate change of treatment. The median effective dose (ED) in millisievert differed significantly (*p* < 0.01) between third-generation dual-source and other CT scanners (1.5 [0.3–9.4] (*n* = 56) vs 3.8 [1.7–20.0] (*n* = 14)).

**Conclusions:**

The diagnostic yield of third-generation dual-source cCTA combined with reduced radiation exposure makes cCTA a favorable diagnostic modality to complete the diagnosis and long-term treatment indications for KD.

**Key Points:**

• *cCTA is a favorable diagnostic modality to complete the diagnosis and long-term treatment indications for Kawasaki disease.*

• *Kawasaki disease patients with proven coronary artery involvement on echocardiography require additional imaging.*

## Introduction

Kawasaki disease (KD) is the most common acquired pediatric heart disease in Western society, which mainly occurs in young children. It is an acute vasculitis where the medium- and small-sized arteries become inflamed. The most precarious complication of this inflammation is the formation of coronary artery aneurysms (CAAs) that can lead to myocardial ischemia and infarction. Identification of patients at risk for ischemia and infarction is essential for treatment optimization and to reduce the morbidity and mortality rate. The American Heart Association (AHA) guidelines recommend risk stratification according to *Z* score (coronary artery diameter corrected for body surface area). The maximal *Z* score combined with the evolution over time (based on echocardiographic findings) has predictive value for the development of myocardial ischemia and infarction. Echocardiography is considered the ideal non-invasive imaging modality for coronary artery assessment. However, even with increased spatial and temporal resolution offered by the newer machines, echocardiography can only detect the proximal sections of the coronary artery tree due to limited ultrasound windows. Notwithstanding this limitation, echocardiography remains the primary imaging modality in the AHA guidelines of 2017, due to its great ease of application in the acute phase of the disease and long-term follow-up in young children.

By recognizing the limitation of echocardiography, the importance of additional imaging for complete coronary artery assessment is emphasized. The most recent AHA guidelines of 2017 suggest that besides echocardiography, angiography (cardiac magnetic resonance imaging [MRI], coronary angiography [CAG], or coronary computed tomographic angiography [cCTA]) should be considered in every patient with CAA (*Z* score > 2.5), but is not routinely applied in clinical practice.

MRI is of additional value to echocardiography and to cCTA mainly for detection of fibrosis, scarring, ischemia, and cardiac function measurement. Because of the invasive nature of invasive CAG and high-radiation exposure of CAG and conventional cCTA, the potential harm often outweighs the benefit. Meanwhile, CT scanners are known to provide high-resolution imaging of the coronary arteries in adults, approaching the quality and accuracy of CAG for detection of coronary aneurysms, which still is the gold standard to date [1]. After the introduction of next-generation CT scanners with low-dose radiation exposure, state-of-the-art imaging by cCTA became also available for children, providing a possible patient-friendly alternative for CAG and a better and more detailed method for coronary artery assessment compared to MRI. Studies have shown this reduction of radiation dose, due to advanced technology and increasing experience that led to better protocols, in the pediatric population over the last years [2, 3].

The aim of this study, consisting of 70 KD patients, was to assess the diagnostic yield of cCTA, compared to echocardiography. The diagnostic yield of cCTA was determined in both the dynamic and static phases of KD (dynamic phase being the subacute and reconvalescent stage up to 2 years after the onset of the disease in which CAA regression can still occur [4–7], static phase being the period thereafter in which coronary artery wall pathology occurs, i.e., calcification, plaque, stenosis). The findings obtained by cCTA were compared with the results of echocardiography to establish the value of additional imaging and to determine the associated radiation dose.

## Methods

### Study population

**Fig. 1:**
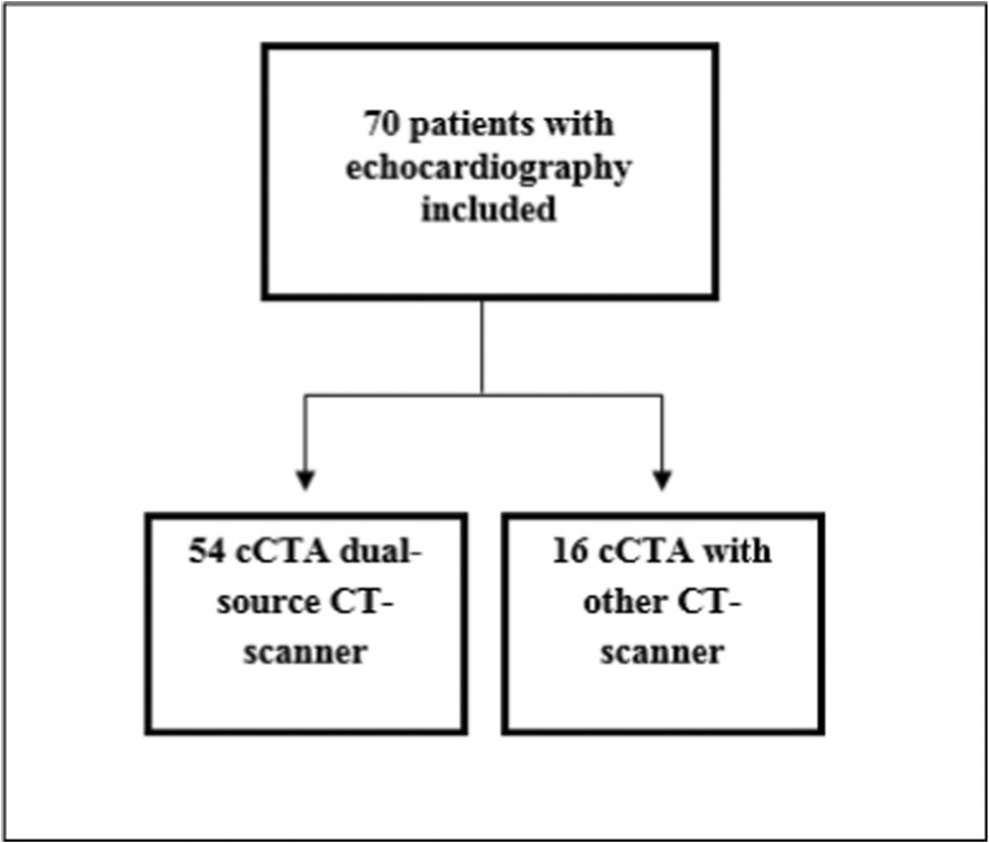
Flow diagram of patient inclusion

KD patients presenting at—or referred to the national referral center for KD and who underwent echocardiography and cCTA—were considered eligible for inclusion in this study. Over the past 8 years (from 2009), 70 consecutive KD patients met the diagnostic criteria of the AHA for KD and underwent cCTA. The cCTA findings were compared to echocardiography (Fig. 1). Clinical information was extracted from medical records for the acute phase and follow-up.

### cCTA

From November 2015, a third-generation dual-source 2 × 192-slice multidetector CT scanner (Siemens SOMATOM Force) was used for cCTA. A prospective ECG-triggered high-pitch spiral scan sequence was used for patients in sinus rhythm and a heart rate below 70 bpm. A prospective ECG-triggered sequential step and shoot sequence was used for patients with an irregular heart rate or heart rate above 70 bpm. The lowest feasible tube voltage was selected by using care-kV, a software tool from Siemens (range 70 to 120 kVp). Intravenous iodine-based contrast medium (Ultravist 300 mg/ml, Bayer Pharmaceuticals) was administered with an injection speed of 0.3–4.0 ml/s depending on body weight (0.5–44 kg) at the antecubital fossa. Scans were analyzed and reported by an experienced cardiovascular radiologist. The results were discussed in a multi-disciplinary team including a radiologist, cardiologist, pediatric cardiologist, and pediatric immunologist (with expertise in KD). Before the introduction of the third-generation CT scanner in 2015, cCTA images were acquired using a 64-slice CT scanner (Philips, Brilliance CT 64 channel) in 9 patients using a prospective ECG-triggered sequential step and shoot sequence and low-pitch spiral scan. In 7 patients, cCTA data from a referring hospital (executed with a 64-slice CT scanner [*n* = 2], 128-slice CT scanner [*n* = 2] and 320-slice CT scanner [*n* = 1] and the third-generation dual-source CT scanner [*n* = 2]) were available for comparison with echocardiography data from our own institution.

### Echocardiography

Echocardiography images were acquired using a GE healthcare (vivid 7, E9, E95) cardiovascular ultrasound system. A trained technician and pediatric cardiologist read all images. Discrepancies were directly compared to avoid misinterpretations in *Z* scores. The technician and pediatric cardiologist were blinded for the cCTA results as the echocardiography was performed before the cCTA. The cCTA and echocardiography were independently analyzed for the maximal diameter in each coronary artery section.

### Measurements

The luminal dimensions of the coronary arteries, i.e., the LMCA (left main coronary artery), LAD (left anterior descending coronary artery), and RCA (right coronary artery), were measured and corresponding *Z* score was reported according to the McCrindle/Boston model; aneurysms were defined as a *Z* score ≥ 3.0. For the circumflex (Cx), *Z* scores are not available. Aneurysms in the Cx were defined when the luminal diameter was ≥ 4.0 mm [5, 8]. In addition, coronary artery lesions, vascular stenosis, vessel wall calcification, intravascular thrombus, and/or plaque formations were registered.

### Effective dose calculation

For analysis of cCTA-associated radiation exposure (in effective dose [ED]), patients were analyzed in 2 groups: third-generation dual-source cCTA and other CT scanners (i.e., 64-slice CT scanner, 128-slice CT scanner, and 320-slice CT scanner). The distinction between the 2 groups was made based on the ability to apply low tube voltages in the range of 70–100 kV. The conversion factors to calculate ED from the dose length product (DLP) were based on pediatric organ and tissue conversion factors from the Commission on Radiological Protection publication 103, available for the ages 0, 1, 5, and 10 years and for adults [9]. In order to calculate the pediatric chest conversion factors for all the ages in our population, we fitted a second-order polynomial curve and interpolated the conversion factor for the missing ages. The conversion factor is dependent on the tube voltage (kVp). Therefore, we also extrapolated the pediatric chest conversion factors for tube voltage of 70 kV and interpolated for the tube voltage of 90 kV.

### Statistical analysis

The radiation exposure in both groups was a non-normal distribution. Therefore, the Mann-Whitney test was performed (IBM Corp. Released 2016. IBM SPSS Statistics for Windows, Version 24.0.).

## Results

### Study population

A total of 70 KD patients were included in our study, of which 78% were male. In this group, 27 patients (38%) had a giant aneurysm (*Z* score ≥ 10) in the acute phase of KD (based on echocardiography results). The median time between the date-of-onset of acute disease and cCTA (ΔTime) was 11.7 years and the median age in years at the time of cCTA was 15.1 (Table 1).

**Table 1:** Demographics of the 70 consecutively included KD patients. CAA status in acute phase was based upon echocardiography results

Demographics	*n* = 70	Remarks
Male	*n* = 55	
Female	*n* = 15	
Age in years at onset KD (median, range)	2.4 (0.12–16.91)	Age-at-onset was unknown in 2 patients.
Missed diagnosis, unknown treatment	*n* = 8	No treatment (IVIG/prednisone) received in 6 cases. Treatment in the acute phase was unknown in 2 others.
Treatment > 10 days after fever onset	*n* = 17	
Treatment day (median, range)	8 (3–34)	Counted from the first day of fever until start of IVIG. In 2 patients, the only information available stated whether IVIG was given within or after 10 days of fever. In 4 patients, the day of treatment was unclear. In 8 patients, IVIG (or prednisone) was not administered.
Non-responder to 1st IVIG	*n* = 18	Persistent fever > 48 h after IVIG treatment.
Age in years at time of CT (median, range)	15.1 (0.48–59.45)	
ΔTime in years (CT date–date of onset) (median, range)	11.7 (0.11–26.00)	Age-at-onset was unknown in 2 cases, which presented at adult age with myocardial infarction (MI).
CAA Z score acute stage: • *Z* score > 10 (giant) • *Z* score 3–10 (small- and medium-sized aneurysms) • *Z* score < 3 (no aneurysm) • Unknown	*n* = 27 *n* = 15 *n* = 26 *n* = 2	

**Fig. 2:**
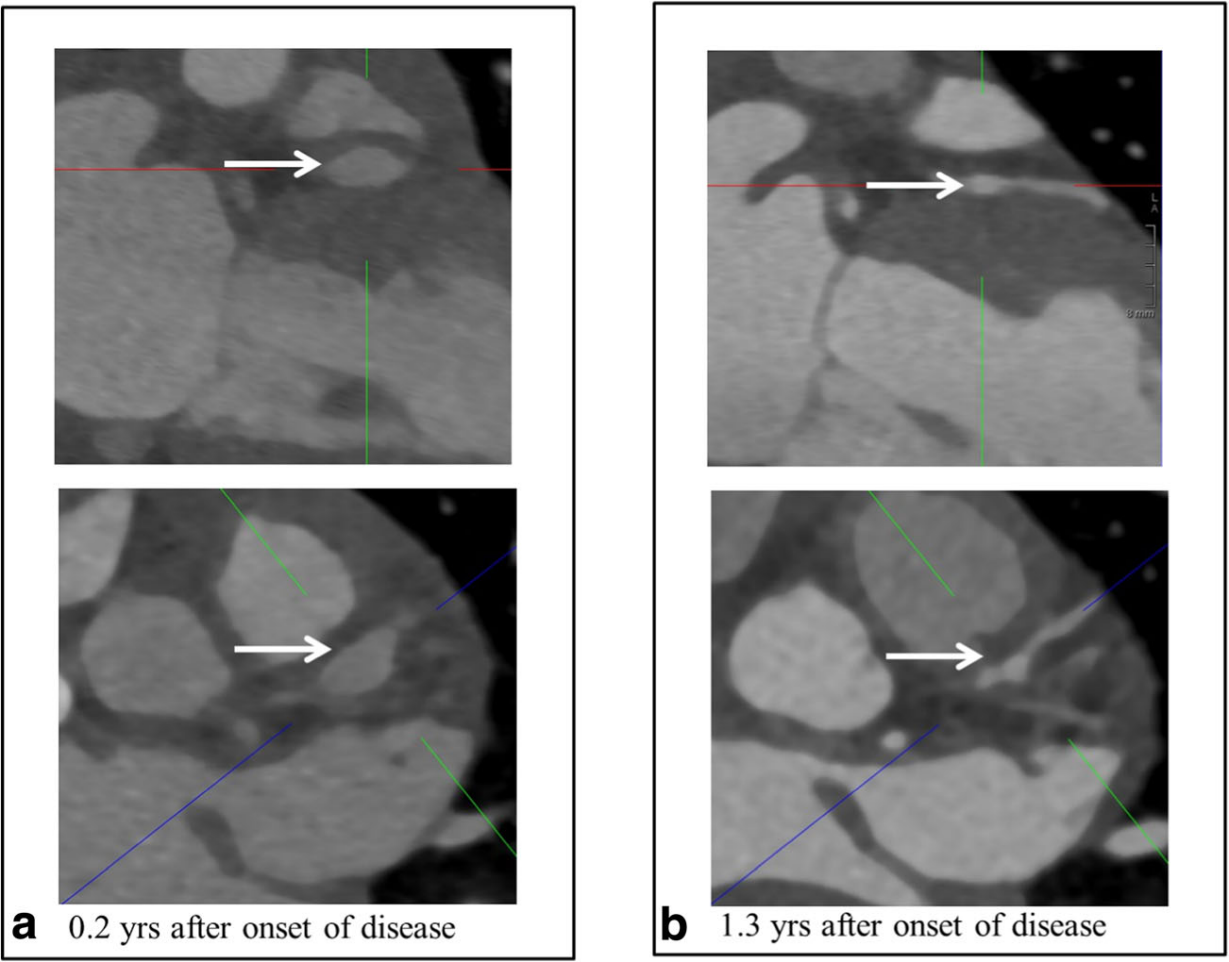
Remodeling of the LAD (in a single patient) performed with the third-generation dual-source CT scanner. **a** Significant aneurysms in the LAD (5.3-mm diameter, *Z* score 16.28). **b** Remodeling of the LAD in the dynamic phase (2.8-mm diameter, *Z* score 4.23)

**Fig. 3:**
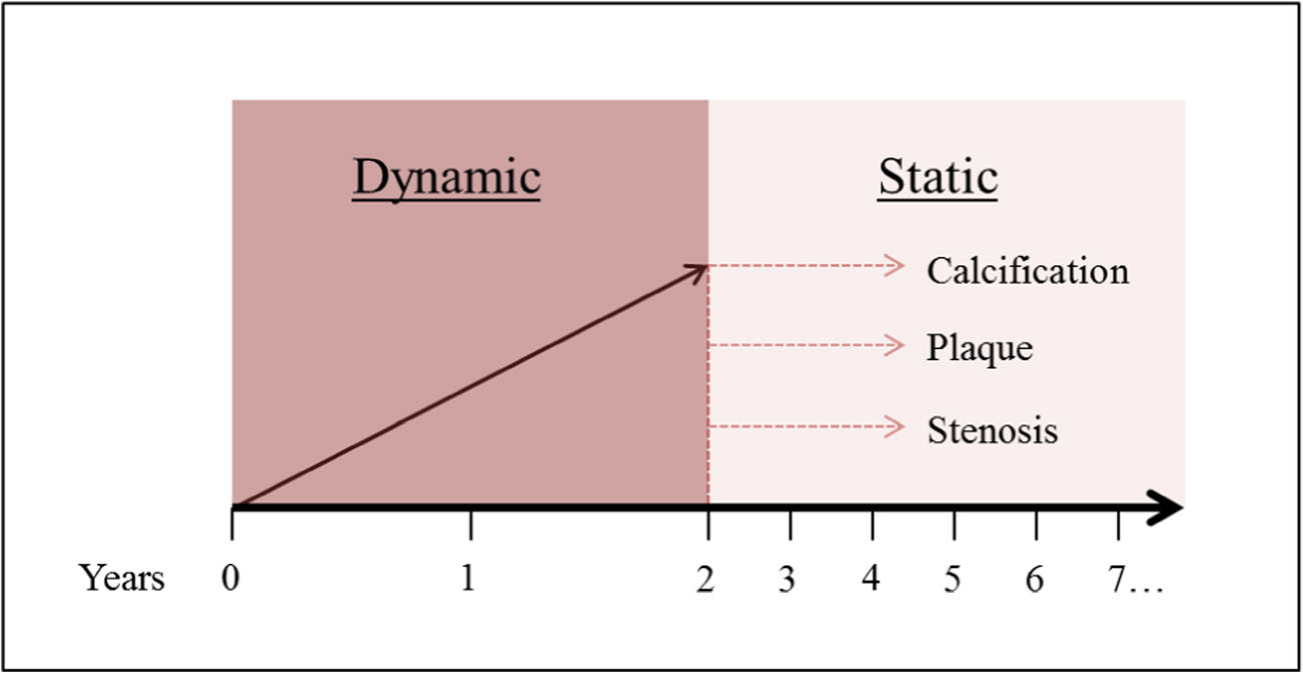
Dynamic and static phase. The years counted being the years after onset of disease. In the first 2 years, the CAAs show most of the remodeling (regression), whereas the years thereafter show secondary complications as calcification, plaque, and stenosis

The imaging in our patients was categorized according to the phase at the time of cCTA. The “dynamic phase” includes the first two years after the onset of disease in which the largest changes in *Z* scores can still occur; an example of this active remodeling has been depicted in Fig. 2, or thereafter in which secondary complications occur: the “static phase” (Fig. 3). The static phase is a stable phase with a view to CAAs.

### cCTA vs echocardiography

In total, 70 cCTAs were performed in the dynamic (*n* = 9) and static phase (*n* = 61), with a respective median time lag (between echocardiography and cCTA) of 31 and 92 days. Without exception, the cCTA acquired images of high-end quality, the images were all of diagnostic quality without disturbing motion artifacts and with adequate luminal attenuation.

**Fig. 4:**
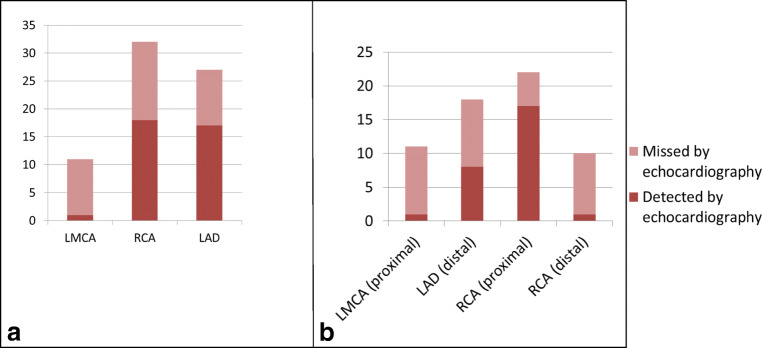
**a** CAAs missed by echocardiography while detected by cCTA. *Y* = number of CAAs. **b** CAAs missed by echocardiography while detected by cCTA. *Y* = number of CAAs

The results were compared to findings obtained by echocardiography performed prior to cCTA. One patient was excluded for the comparative analysis because of prior coronary artery bypass grafting (CABG) and unreliable echocardiography at the time of cCTA. We found discrepant results between echocardiography and cCTA. In total, we observed 61 CAAs with the cCTA, whereas only 27 CAAs had been visualized by echocardiography. Echocardiography was unable to detect 34 CAAs (56%), in the LMCA (*n* = 10), RCA (*n* = 14), and LAD (*n* = 10) (Fig. 4a, Table 2). Of these 34 undetected CAAs, 5 CAAs (in 4 patients) were detected during the dynamic phase in the LMCA (*n* = 1), RCA (*n* = 3), and LAD (*n* = 1).

**Table 2:** Overview of all cCTA performed (*n* = 69) compared to echocardiography (*n* = 69)

Coronary artery	CAA by cCTA (no. of patients)	CAA by echocardiography (no. of patients)	Not visualized by cCTA (not interpretable) (no.)	Not visualized by echocardiography (not interpretable) (no.)
LMCA	11 (11)	1 (1)	0	2
RCA	32 (22)	18 (17)	0	1
LAD	18 (14)	8* (7*)	0	49**

*Does not include one CAA that was depicted by echocardiography in the dynamic phase which was not depicted by cCTA due to remodeling (Fig. 2)

**Out of 49 echocardiographs in which the LAD was not visualized, 8 cCTAs identified a total of 9 CAAs

#### Proximal segments

In 15 (44%) of the abovementioned 34 “missed CAAs,” the proximal segments of the coronary arteries were correctly identified by both imaging methods, but a difference in the luminal dimension of the CAA measurement became apparent. cCTA defined these lesions as abnormal by size and hence falling in the category of CAA (taking a *Z* score ≥ 3 as abnormal), while defined shortly before as “normal” upon echocardiography (median time between echocardiography and cCTA was 73.5 days) (Fig. 4b).

#### Distal segments

As expected, the echocardiography was prone to miss CAAs in the distal segments that were difficult or impossible to visualize. In 19 (55.8%) of the before-mentioned 34 “missed CAAs,” the CAAs were missed due to the distal location. In the most commonly affected coronary arteries, i.e., the RCA and LAD [7], we found that a 2nd or 3rd CAA in the RCA was often missed. In the RCA, cCTA identified a total of 32 CAAs in 22 patients whereas a total of 18 CAAs in 17 patients was detected by echocardiography. Echocardiography was also prone to miss CAAs in the other most commonly affected coronary artery, the LAD: i.e., cCTA showed a total of 18 CAAs in 14 patients whereas echocardiography only identified a total of 8 CAAs in 7 patients (Fig. 4b). This was mainly caused by the fact that echocardiography was not able to visualize the LAD 49 times of the cases, where cCTA was able to localize 9 CAAs in the LAD (Table 2).

#### Additional findings

In our study population, the circumflex artery (Cx) was not detected by echocardiography, whereas the Cx was visualized in all patients by cCTA. Because *Z* scores are based on echocardiography findings, there are no normal values for the luminal diameter of the Cx available. Despite this drawback, in 6 patients (8.6%), the Cx was considered to be enlarged with a luminal diameter ≥ 4 mm by cCTA or an internal diameter of a segment measuring 1.5 to 4 times that of an adjacent segment at the age of ≥ 5 years, including 1 giant CAA (Table 3).

**Table 3:** Aneurysmatic lesions in the Cx measured by cCTA. Lesions of > 4 mm but ≤ 8 mm at the age of ≥ 5 years were identified in 5 patients. In 1 patient, a true giant CAA (diameter of > 8 mm, qualifying as a giant  according to the Japanese guidelines) was observed

Cx in mm	Adjacent segment in mm	Age at time of cCT in years
7.4	1.8	12.5
4*	2.2	8.0
9.5	2.6	30.1
6.0	3.0	18.0
4.2	2.7	15.0
7	3.7	59.5

*The Cx was overall dilated

### Coronary artery pathology

**Fig. 5:**
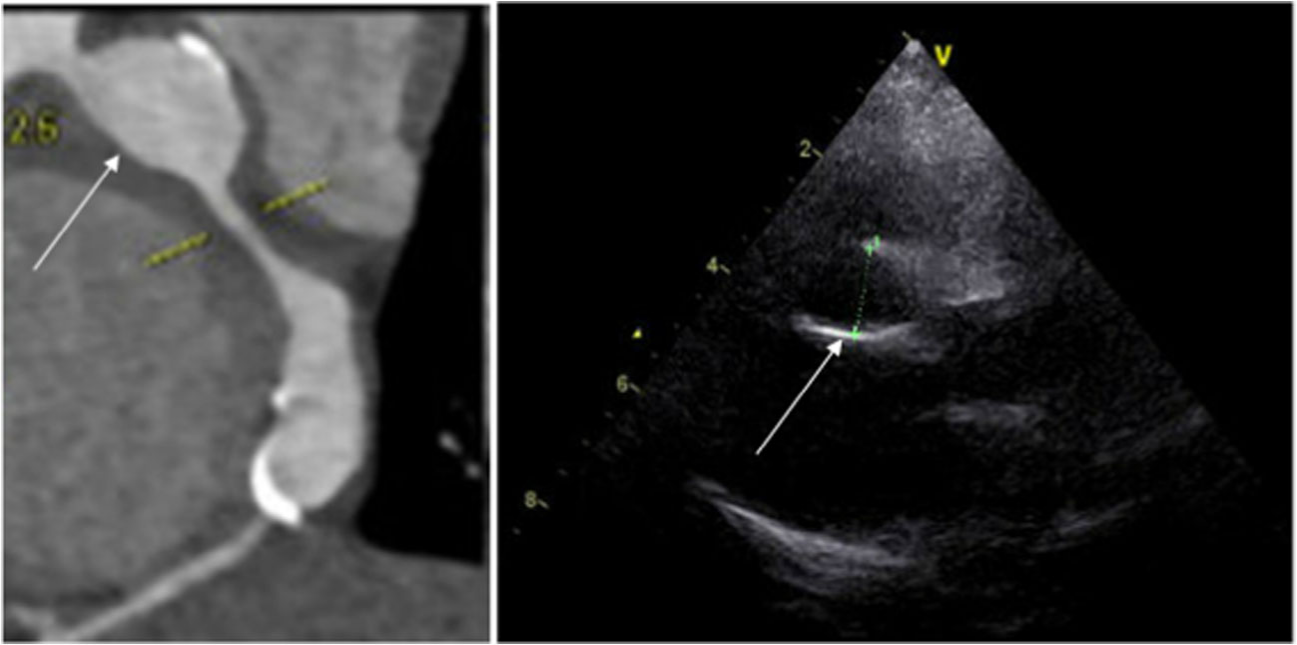
Proximal and distal aneurysm in the RCA with calcification depicted by cCTA vs echocardiography

In 15 patients, additional pathological features consisting of calcification (*n* = 13) depicted in Fig. 5 (cCTA vs echocardiography), luminal stenosis (*n* = 6), thrombosis (*n* = 5), and plaque formation (*n* = 4) were identified (Table 4). Out of the 13 patients with calcified coronary arteries upon cCTA, calcifications were registered at ΔTime of < 10 years in 3 children (at 2.7, 3.1, and 6.1 years, respectively). These 3 patients had a maximal *Z* score (between 12.3 and 39.6) during the dynamic phase of the disease at the same location in these coronary arteries, accentuating the severe course of the coronary disease during their acute stage. In 2 of these 15 patients, a relevant change in clinical management was based on the cCTA findings: i.e., invasive CAG with dilation of the stenosis (*n* = 1) and the start of cholesterol-lowering medication due to the combination of early calcification, obesity, and hyperlipidemia (*n* = 1).

**Table 4:** Overview of coronary artery pathology visualized by cCTA. ΔTime = CT date − date of onset

ΔTime in years	LMCA	RCA	LAD	Cx	Acute stage *Z* score
12.2	Calcification	Calcification	None	None	*Z* score > 10 (giant)
3.1	None	Calcification, thrombus	Calcification, thrombus	None	*Z* score > 10 (giant)
16.8	None	Stenosis (occlusion)	None	None	*Z* score > 10 (giant)
> 2	None	Calcification	Calcification, stenosis	None	Unknown
11.7	None	None	None	Calcification	*Z* score > 10 (giant)
13.0	none	Calcification, soft plaque, stenosis	None	None	*Z* score > 10 (giant)
2.5	None	None	Thrombus	None	*Z* score > 10 (giant)
2.7	None	Calcification, stenosis	Calcification, stenosis	None	*Z* score > 10 (giant)
6.1	None	Calcification	None	None	*Z* score > 10 (giant)
18.5	None	Stenosis (occlusion)	Stenosis (occlusion)	Calcification	*Z* score > 10 (giant)
18.2	None	Calcification, thrombus	Calcification	None	*Z* score > 10 (giant)
23.3	None	Calcification	Calcification, stenosis (occlusion)	None	*Z* score > 10 (giant)
12.7	None	Calcification and plaque	None	None	*Z* score 8.10
> 2	None	Calcification	Calcification, sclerosis	None	*Z* score > 10 (giant)
13.0	None	Thrombus, calcification	Plaque and possible stenosis	None	*Z* score > 10 (giant)

### Radiation exposure

**Fig. 6:**
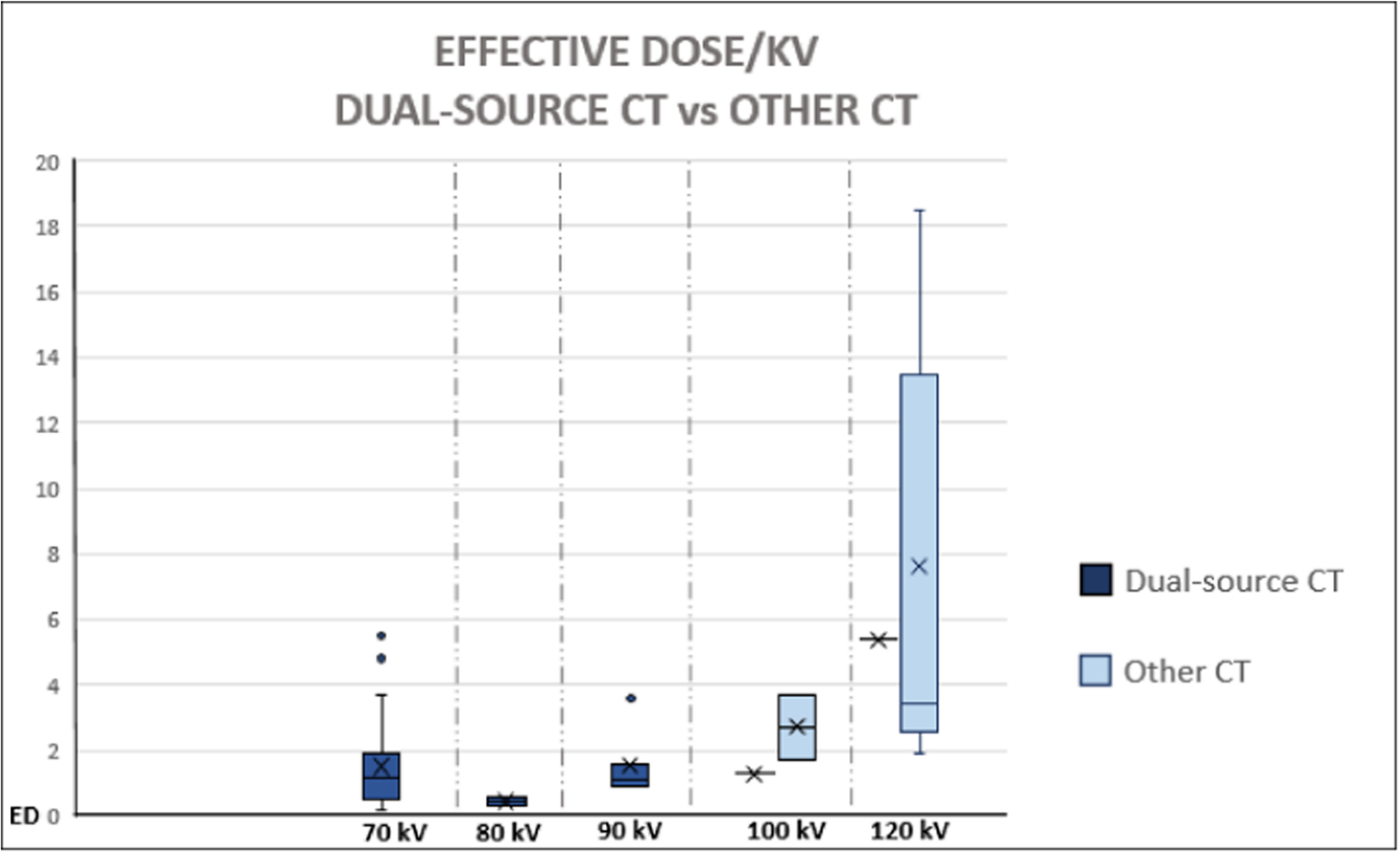
Effective dose (in mSv) with tube voltage (kV) used in the same acquisition in order to depict the coronary arteries. Third-generation dual-source CT scanner vs other CT scanners. For the other CT scanners, there were no tube voltages (kVp) of 70 kV, 80 kV, or 90 kV in these acquisitions

Of the 70 cCTAs performed and analyzed in the study, a total of 56 scans had been executed with a third-generation dual-source CT scanner. The median cumulative ED (in millisievert [mSv]) for this CT scanner was 1.5 mSv (range 0.3–9.4 mSv; *n* = 56), whereas in other CT scanners, the ED was 3.8 mSv (range 1.7–20.0 mSv; *n* = 14); this difference is statistically significant (*p* < 0.01). The wide range in ED of the third-generation dual-source cCTA group was due to several reasons, i.e., (1) additional scan for calcium scoring in 6 cases (which was abandoned as additional sequence after these first 6 cases because of the lack of extra information obtained), (2) adaptation of scan mode from high-pitch spiral to sequential because of an elevated and/or irregular heart rate in 16 cases, and (3) a second/additional acquisition needed in 2 cases because of a suboptimal contrast timing or motion artifacts. The radiation exposure (ED) used for the scan that acquired the images of the coronary arteries in relation to the tube voltage (kVp) is depicted in Fig. 6; the tube voltage (kVp) had a range of 70 to 120 kV, with an average of 97 kV.

## Discussion

State-of-the-art cCTA with low radiation dose for children and young adults has a high diagnostic yield for coronary artery assessment, which makes the third-generation dual-source cCTA a favorable diagnostic imaging modality in the diagnosis of KD and should be considered complementary to echocardiography.

Several case series reported the diagnostic accuracy of cCTA for the assessment of coronary arteries in KD when compared to CAG [10, 11] and echocardiography [12–14]. However, only 3 small studies have reported the additional diagnostic yield of third-generation dual-source cCTA compared to echocardiography. The first study described 19 KD patients of which 15 had CAAs and the second study included 24 KD patients of which all had CAAs [15, 16]. The third study described 12 KD patients at time of initial diagnosis [17]. One study compared dual-source cCTA to CAG in a group of 25 KD patients of which all had CAAs [18]. Although small study groups were used, all 4 studies agree in their conclusion that dual-source cCTA is a reliable and accurate technique for the assessment of the coronary arteries in KD.

In the current study, a total of 70 cCTAs in KD patients were compared to echocardiography findings. cCTA was found to detect twice the amount of CAAs compared to echocardiography, confirming the limitations of echocardiography as shown in previous studies with smaller series and using CT [12, 14, 15]. This study adds information about the high sensitivity of cCTA for detection of CAAs in KD and about the distribution of the previously missed CAAs. The majority of CAAs that were missed by echocardiography were located in the LAD and in more distal segments of the RCA. Moreover, cCTA was able to detect CAAs in the Cx where echocardiography is technically not able to visualize the artery properly.

In 14 patients (20%), the cCTA findings were relevant for CAA classification, i.e., no CAA, small CAA, medium CAA, or giant CAA. This led in only 3 patients to (re)start treatment whereas in the remaining patients oral therapy was simply advised to continue; this as a result of our awareness of the limitations of echocardiography and our precautious approach in translating echocardiography results into clinical practice. Furthermore, 15 patients had additional coronary artery pathology upon cCTA that was not detected by echocardiography, including thrombosis, stenosis, plaques, and calcification. In 2 of these 15 patients, a change in clinical management was based on these cCTA findings.

Some of the consequences of the cCTA findings in the patients with a CAA that had not been detected by echocardiography at all (distal segments) are to be considered. First, it resulted in clear re-categorization of their CAA status (using the proximal segments as detected by echocardiography). There were no isolated CAAs in the distal segments without involvement of the proximal segment, but the diameter of the distal CAA could exceed the diameter of the proximal CAA. In fact, in 14 out of the 69 patients (20%), despite multiple CAAs, the cCTA findings were of relevance for the CAA scoring as such (*Z* score < 3 changed to small/medium-sized CAA-positive [*n* = 11] and giant CAA [*n* = 2]), and a reinterpretation of a medium-sized to a giant CAA (*Z* score > 10 [*n* = 1]). Of the 11 patients that changed from “no CAA” to “small/medium-sized CAA” 2 were performed in the dynamic phase, which still need further follow-up.

Echocardiography remains the primary imaging modality. *Z* scores are based on the deviation of a normal distribution of luminal diameters in healthy children as measured by echocardiography and have their limitations. First, *Z* scores are based on luminal diameters in children and not validated in (young) adults, which are much more difficult if not impossible to undergo routine coronary echocardiography. Secondly, the Cx is not visualized by echocardiography. The absence of *Z* scores for the Cx does not imply that there are no aneurysms in the Cx. In fact, we found in 6 patients an enlarged Cx (≥ 4 mm), based on the Japanese Circulation Society (JCS) that existed before the *Z* scores were defined. Based on these criteria, one of the aberrant Cx would be classified as a giant aneurysm with a luminal diameter of 9.5 mm (as JCS classifies a luminal diameter ≥ 8 as a giant aneurysm) [19]. As a result, altered hemodynamics could cause occlusion or thrombosis of the Cx and infarction of the posterior and posterolateral wall of the left ventricle if left untreated with anticoagulants.

These results emphasize the necessity of additional imaging besides echocardiography. Whereas before the MRI was considered a feasible and accurate method to identify coronary artery pathology in addition to echocardiography [20], studies reported ambiguous diagnostic value in the detection of small aneurysms [20–24]. Moreover, cCTA has undergone tremendous technical advancements in the recent years, which made it possible to require high-quality images even at high heart rates with highly acceptable radiation exposure. This makes the third-generation dual-source cCTA an excellent and better candidate for detailed coronary artery diameter and vessel wall assessment, especially in pediatric populations. A reason to (initially) refrain from cCTA during follow-up and chose MRI as a first step consists of extreme anxiety in the pediatric patient for intravenous administration of contrast agent, previous allergic reactions to contrast agents, or suspected cardiac ischemia for the detection of fibrosis, scarring, ischemia, and cardiac function measurement.

The timing to perform early cCTA following acute disease will strongly depend on the clinical presentation but seems justified to be performed after 2–3 months when increasing diameter has become less likely and before regression occurs [5].

### Limitations

This is a retrospective study with inherent limitations, of which selection bias seems the most obvious limitation. Our study population is not a mere representation of the normal KD population due to the fact that the patients were selected because of the suggested necessity of imaging (often with CAAs at present or in the past) in addition to standard echocardiography according to the AHA 2004 and 2017 guidelines. For that reason, the study population does not reflect the epidemiological patterns in the general KD population.

## Conclusion

Third-generation dual-source cCTA has a good diagnostic yield compared to echocardiography. In 22 (32%) out of 69 individuals with KD in the past, additional coronary lesions were observed, which had not been detected by echocardiography. In addition, 6 aneurysms in the left circumflex were found and coronary artery changes occurred much earlier than previously has been reported. With the third-generation dual-source CT scanners, radiation dose is significantly reduced. This lower radiation exposure makes the benefits counterbalance the potential harm and thereby enables accurate coronary artery assessment in KD by cCTA in a routine clinical setting. The third-generation dual-source cCTA is a safe and accurate standard complementary imaging modality in addition to echocardiography in all KD patients—in particular, those with CAA *Z* score ≥ 3—and clearly adds to the interpretation of the coronary status.

## Abbreviations

AHA: American Heart Association
CAA: Coronary artery aneurysm
CABG: Coronary artery bypass grafting
CAG: Coronary angiography
cCTA: Coronary computed tomographic angiography
Cx: Circumflex artery
DLP: Dose length product
KD: Kawasaki disease
LAD: Left anterior descending coronary artery
LMCA: Left main coronary artery
MRI: Magnetic resonance imaging
mSv: Millisievert
RCA: Right coronary artery
