# Correction to: Coronary artery assessment in Kawasaki disease with dual-source CT angiography to uncover vascular pathology

**DOI:** 10.1007/s00330-021-08262-5

**Published:** 2021-10-20

**Authors:** D. van Stijn, R. N. Planken, M. Groenink, G. J. Streekstra, T. W. Kuijpers, I. M. Kuipers

**Affiliations:** 1 Department of Pediatric Hematology, Immunology and Infectious Diseases, Emma Children’s Hospital, Amsterdam UMC, University of Amsterdam, Amsterdam, The Netherlands; 2 Department of Radiology and Nuclear Medicine, Amsterdam UMC, University of Amsterdam, Amsterdam, The Netherlands; 3 Department of Cardiology, Amsterdam UMC, University of Amsterdam, Amsterdam, The Netherlands; 4 Department of Pediatric Cardiology, Emma Children’s Hospital, Amsterdam UMC, University of Amsterdam, Amsterdam, The Netherlands

**Correction to: European Radiology (2020) 30:432–441**

10.1007/s00330-019-06367-6

The original version of this article, published on 19 August 2019, unfortunately contained a mistake. The following correction has therefore been made in the original: The author name D. van Stijn–Bringas Dimitriades was changed to D. van Stijn. The corrected author list is given above. The original article has been corrected.

